# Transgenic Expression of Human Lysophosphatidic Acid Receptor LPA_2_ in Mouse Intestinal Epithelial Cells Induces Intestinal Dysplasia

**DOI:** 10.1371/journal.pone.0154527

**Published:** 2016-04-28

**Authors:** Michihiro Yoshida, Peijian He, C. Chris Yun

**Affiliations:** 1 Division of Digestive Diseases, Department of Medicine, Emory University, Atlanta, Georgia United State of America; 2 Department of Gastroenterology and Metabolism, Nagoya City University Graduate School of Medical Sciences, Nagoya, Japan; 3 Winship Cancer Institute, Emory University, Atlanta, Georgia, United State of America; Cincinnati Children's Hospital Medical Center, UNITED STATES

## Abstract

Lysophosphatidic acid (LPA) acts on LPA_2_ receptor to mediate multiple pathological effects that are associated with tumorigenesis. The absence of LPA_2_ attenuates tumor progression in rodent models of colorectal cancer, but whether overexpression of LPA_2_ alone can lead to malignant transformation in the intestinal tract has not been studied. In this study, we expressed human LPA_2_ in intestinal epithelial cells (IECs) under control of the villin promoter. Less than 4% of F1-generation mice had germline transmission of transgenic (TG) human LPA_2_; as such only 3 F1 mice out of 72 genotyped had TG expression. These TG mice appeared anemic with hematochezia and died shortly after birth. TG mice were smaller in size compared with the wild type mouse of the same age and sex. Morphological analysis showed that TG LPA_2_ colon had hyper-proliferation of IECs resulting in increased colonic crypt depth. Surprisingly, TG small intestine had villus blunting and decreased IEC proliferation and dysplasia. In both intestine and colon, TG expression of LPA_2_ compromised the terminal epithelial differentiation, consistent with epithelial dysplasia. Furthermore, we showed that epithelial dysplasia was observed in founder mouse intestine, correlating LPA_2_ overexpression with epithelial dysplasia. The current study demonstrates that overexpression of LPA_2_ alone can lead to intestinal dysplasia.

## Introduction

Colorectal cancer (CRC) is one of the leading causes of cancer-related deaths worldwide. CRC develops through a series of genetic modifications that transform normal colonic epithelium to an adenoma and the adenocarcinoma. Among the earliest events in the progression of CRC is loss of the *APC* gene or activating mutation of β-catenin. Subsequent genetic alterations in *Kras* gene, 18q loss of heterozogosity, *Smad4*, and *p53* lead to adenoma-to-carcinoma progression [1]. In addition to the genetic instability, the activation of growth factor pathways is common in the pathogenesis of CRC cells. The activation of Cox-2, epidermal growth factor, and vascular endothelial growth factor is associated with various stages of CRC development and the extent of disease progression [2].

Lysophosphatidic acid (LPA) is a small glycerolipid acting on a family of G-protein-coupled receptor, LPA_1_-LPA_6_ and induces growth factor-like effects that regulate cell proliferation, survival, migration, and secretion of cytokines [3–5]. Among the LPA receptors, elevated expression levels of LPA_2_ suggest a considerable pathophysiological relevance to carcinogenesis [6–9]. LPA_2_ expression is increased in CRC patients and proportionally increases with the size of adenomas in Apc^Min/+^ mice [9, 10]. LPA_2_ was shown to protect intestinal epithelial cells (IECs) from chemotherapeutics-induced apoptosis and radiation-induced damage [11, 12]. In addition, The LPA-LPA_2_ signaling axis promotes tumor cell proliferation through the activation of transcriptional factors, such as β-catenin, Kruppel-like factor 5 (KLF5), NF-κB, and hypoxia-inducible factor 1α (HIF-1α) [13–17]. The expression levels of LPA_2_ correlates with increased sizes of adenomas in Apc^Min/+^ mice, and loss of LPA_2_ decreased carcinogen-induced colon cancer and a mouse model of familial adenomatous polyposis, Apc^Min/+^ [10, 18].

Elevated expression of LPA_2_ in several cancers has suggested a causative relationship between LPA and cancer, but it still remains unclear to what extent LPA_2_ contributes to cancer development and progression. Overexpression of LPA receptors as well as LPA-producing autotaxin in mouse mammary gland resulted in invasive and metastatic mammary cancer [19]. On the other hand, LPA_2_ overexpression in mouse ovaries did not form spontaneous tumor albeit elevated vascular endothelial growth factor (VEGF) and urokinase-type plasminogen activator levels compared to non-transgenic ovaries [20]. The goal of this study is to determine whether increased LPA_2_ expression in IECs alone is sufficient to induce spontaneous transformation in the intestinal tract.

## Materials and Methods

### Generation of LPA_2_ transgenic mice

Transgenic (TG) mice overexpressing human LPA_2_ in IECs were generated by the Emory Transgenic Core facility. In brief, human LPA_2_ with a VSVG-tag fused at the N-terminus was cloned into the MluI/BsiWI sites of pBSKS Villin MES vector [21]. The transgene DNA was excised with SalI, purified, and injected into the pronuclei of fertilized eggs of C57BL/6J mice. TG founder (F0) mice were identified by PCR analysis of tail genomic DNA with two sets of primer pairs. Primer pair A: forward 5’-ATGAACCGCCTGGGTAAGAT-3’ and reverse 5’-GCCGAGCAGGTAGTAGATGG-3’; primer pair B: forward 5’-TTGTCTTCCTGCTCATGGTG-3’ and reverse 5’-CTCGGCAAGAGTACACAGCA-3’. Stable LPA_2_ TG mice (F1) were obtained by crossing founder mice with wild type C57BL/ 6J mice. Mice were housed in individually ventilated cages maintained at 20–22°C with constant humidity (40 ± 10%), a 12 h light-dark cycle, and free access to regular chow and water. Mice were monitored daily for clinical signs including weight loss, food and water intake, and activity levels. Newly born mice were genotyped between 8 and 12 days of age. Transgenic F1 mice were smaller in size and appeared frail. F1 mice were monitored twice daily by the investigators, and resident veterinarians were consulted to postpone weaning by one week to have nursing supports from their mother. After weaning, F1 mouse was provided with additional supplemental hydration and nutrient gels. Nonetheless, the mouse had poor feeding without a significant increase in body weight. The mouse had a low level of physical activity and appeared lethargic, requiring euthanasia. All animals were euthanized by CO_2_ asphyxiation followed by cervical dislocation. All efforts were made to minimize animal suffering. All animal experiments were carried out under approval by the Institutional Animal Care and Use Committee of Emory University and in accordance of the NIH Guide for the Care and Use of Laboratory Animals.

### Antibodies

Mouse anti-VSVG polyclonal antibody and rabbit anti-NHE3 polyclonal antibody were previously described [22]. Rabbit anti-KLF5 polyclonal antibody was a kind gift of Dr. Jonathan Katz (Univ. of Pennsylvania). Rabbit polyclonal anti-Ki67 antibody was obtained from Leica (Newcastle, UK). Rabbit monoclonal anti-cleaved caspase-3 antibody was obtained from Cell Signaling Technology (Danvers, MA). Rabbit monoclonal anti-lysozyme antibody and rabbit polyclonal anti-chromogranin (CgA) antibody were obtained from Abcam (Cambridge, MA).

### Immunohistochemistry (IHC)

Immunohistochemical analysis was performed as previously describe with modifications [23]. Mouse intestine sections were flushed with cold PBS, and were fixed overnight in 10% buffered formalin. Intestinal tissues were paraffin-embedded, and sectioned at 4 μm. Tissue sections were deparaffinized and dehydrated in a graded series of xylene and ethanol. Antigen retrieval was achieved by heating the samples in 10 mM citrate buffer, pH 6.0 with 0.05% Tween 20, in a cooker for 10 min at 125°C. After inhibition of endogenous peroxidase activity by immersion in 3% H_2_O_2_/methanol solution, tissue sections were blocked with goat serum, and incubated with primary antibody overnight at 4°C. After 3 washes in PBS, sections were incubated with biotinylated secondary antibody and then with avidin-biotin horseradish peroxidase solution (Vector Laboratories, Burlingame, CA). Finally, tissue sections were incubated with 0.01% H_2_O_2_ and 0.05% 3,3'-diaminobenzidine tetrachloride. Nuclear counterstaining was accomplished using Mayer’s hematoxylin.

### Confocal immunofluorescence

Sections were deparaffinized and rehydrated, and antigen unmasking was performed as above. The fluorescence staining procedures were described previously [23]. Briefly, tissue sections were permeated with PBS containing 0.2% Triton-X 100 for 10 min, followed by washes. Then tissue sections were blocked with goat serum, and incubated with anti-VSVG polyclonal antibody for 1 h at room temperature. After washes with PBS, sections were incubated with Alexa Fluor 488-conjugated goat anti–mouse immunoglobulin G (IgG) and Hoechst 33342 for 30 min at room temperature. After washes with PBS, the specimens were mounted with ProLong Gold Antifade Reagent (Invitrogen) and observed under a Zeiss LSM510 laser confocal microscope (Zeiss Microimaging, Thornwood, NY).

### Ki67 and cleaved caspase-3 staining

Rabbit polyclonal anti-Ki67 antibody and rabbit monoclonal anti-cleaved caspase-3 antibody were used for staining as described above. Proliferating cells were quantified by counting Ki67-positive cells per crypt. Thirty crypts were quantified in each mouse. The level of apoptosis was expressed as percentage of cleaved caspase-3-positive cells over total number of cells within a field of vision at a magnification of x200. A total of twenty fields were scanned for each mouse.

### Intestinal alkaline phosphatase staining

Tissue sections were stained for endogenous intestinal alkaline phosphatase activity using Vulcan Fast Red Chromogen kit (Biocare Medical), following manufacturer's recommendations.

### Alcian Blue staining

Deparaffinized tissue sections were incubated in 3% glacial acetic acid solution for 3 minutes, followed by 20 min incubation in alcian blue solution (1% alcian blue in 3% glacial acetic acid, pH2.5). Sections were counter stained with Nuclear Fast Red (Sigma) for 1 minute. Goblet cells were quantified by counting alcian blue positive cells in cross-sectional views of 30 intestinal villi or 30 colonic crypts per each mouse.

### Statistical analysis

Statistical significance was assessed by two-tailed unpaired Student’s *t* test. Values are expressed as mean ± standard error of mean (SEM). A *P* value of < 0.05 was considered statistically significant.

## Results

### Generation of LPA_2_ transgenic mice

**Fig 1:**
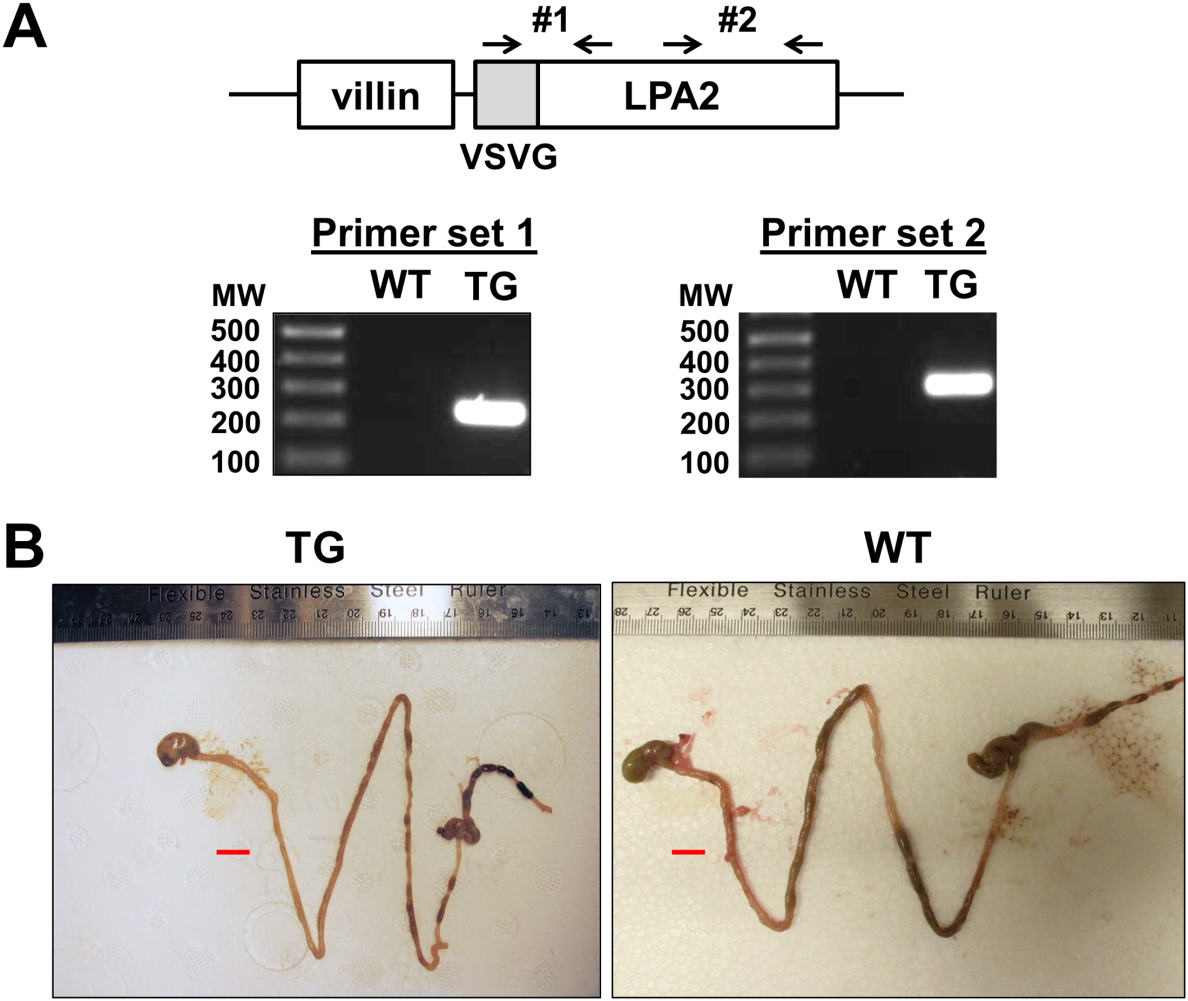
Generation of LPA_2_ TG mice. **(A)** Schematic representation of LPA_2_ TG construct and genotyping of TG mice using two sets of primers are shown. **(B)** The entire intestinal tract of LPA_2_ TG and WT mice are shown. Bar: 1 cm.

Transgenic mice were generated using the pBS-KS Villin-LPA_2_ construct, which contains human LPA_2_ gene fused with a VSVG tag at the N-terminus. Seven transgenic founder (F0) mice were identified by PCR analysis from four independent microinjections into oocytes of C57BL/6J mice (Fig 1A). Founder mice were backcrossed to C57BL/6J mice to generate progeny. Out of 72 offsprings, only three mice, 2 males and 1 female, showed germline transmission of the transgene. Two of the three F1 transgenic mice died at the age of 15 days and the remaining mouse was small in size compared with the wild type mouse of the same age and sex, and appeared anemic with hematochezia. Despite the provision of moisturized chow and gelpacs, the body weight of the transgenic mouse was only 8.1 g at the age of 35 days compared to an average weight of 19 g for WT C57BL/6J mice. The transgenic mice were euthanized according to the guideline by the Emory University Animal Case and Use Committee.

### LPA_2_ transgenic mice show dysplasia in the intestine

**Fig 2:**
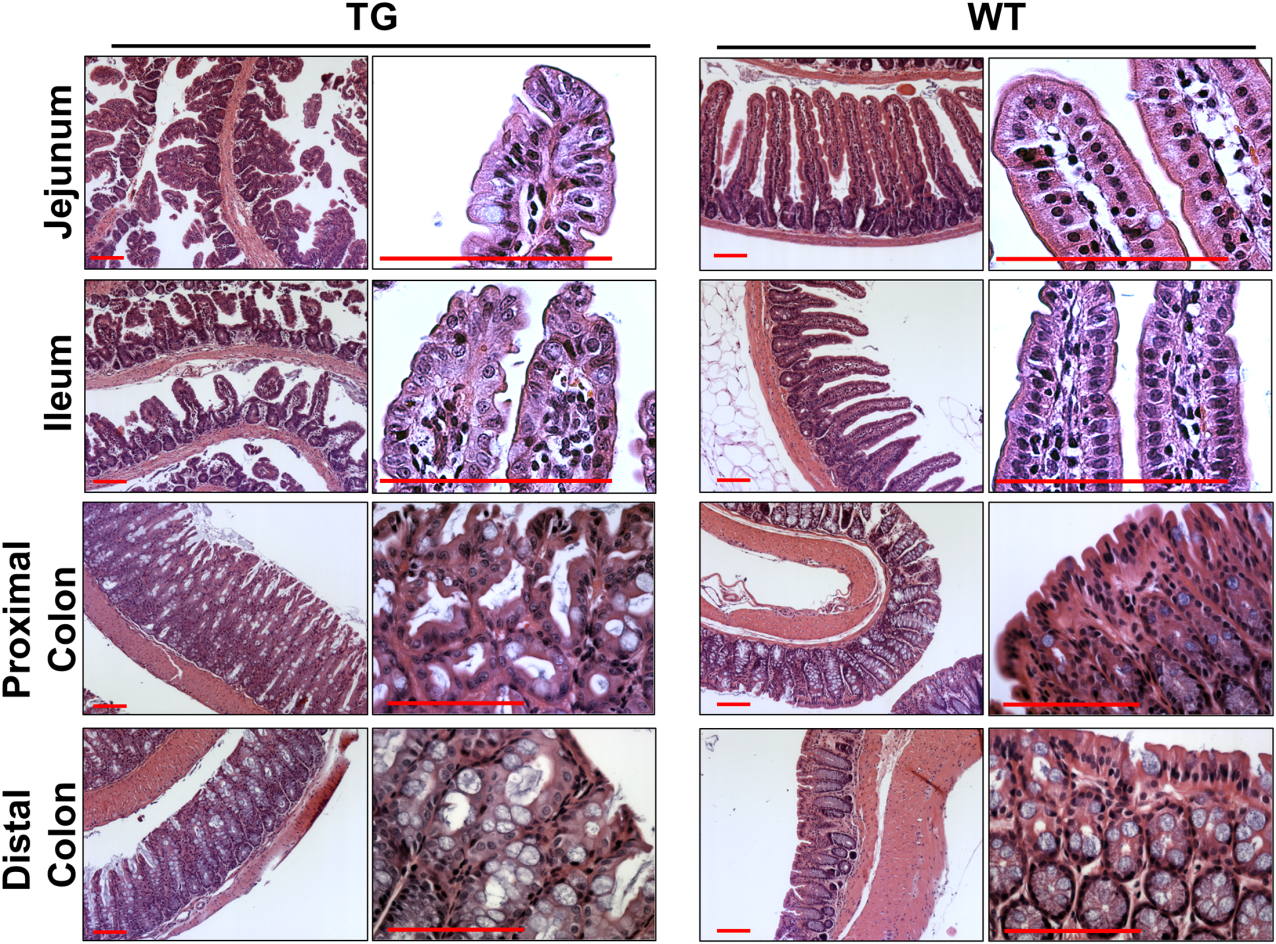
TG expression of LPA_2_ in the intestinal tract results in dysplasia. H&E staining of small intestine (jejunum and ileum) and colon (proximal and distal) of LPA_2_ TG and WT mice are shown. Magnification: x100. Right panels show high power images. Bar: 100 μm. n = 3 per group.

Comparison of the entire intestinal tract of the transgenic mice with WT counterparts showed that the luminal diameter of the intestinal tract was smaller in the TG mice than WT control although the length was similar. In addition, blood clots were found posterior to cecum consistent with the presence of hematochezia (Fig 1B). Histological analysis showed that the intestinal villi were disarranged and blunted throughout the small intestine. (Fig 2) The epithelial cells were dysplastic with shorter length. However, the crypt density was not significantly different compared with WT mice. Surprisingly, the alteration in TG colon was markedly different from that of TG small intestine. There was an increase in colonic crypt depth, and the effect was more prominent in the proximal than distal part of the colon. Higher magnification view of the TG colon revealed that there were hyperplatic changes with mild dysplasia. However, the overall extent of dysplasia in the distal colon was relatively low and was not consistently observed.

**Fig 3:**
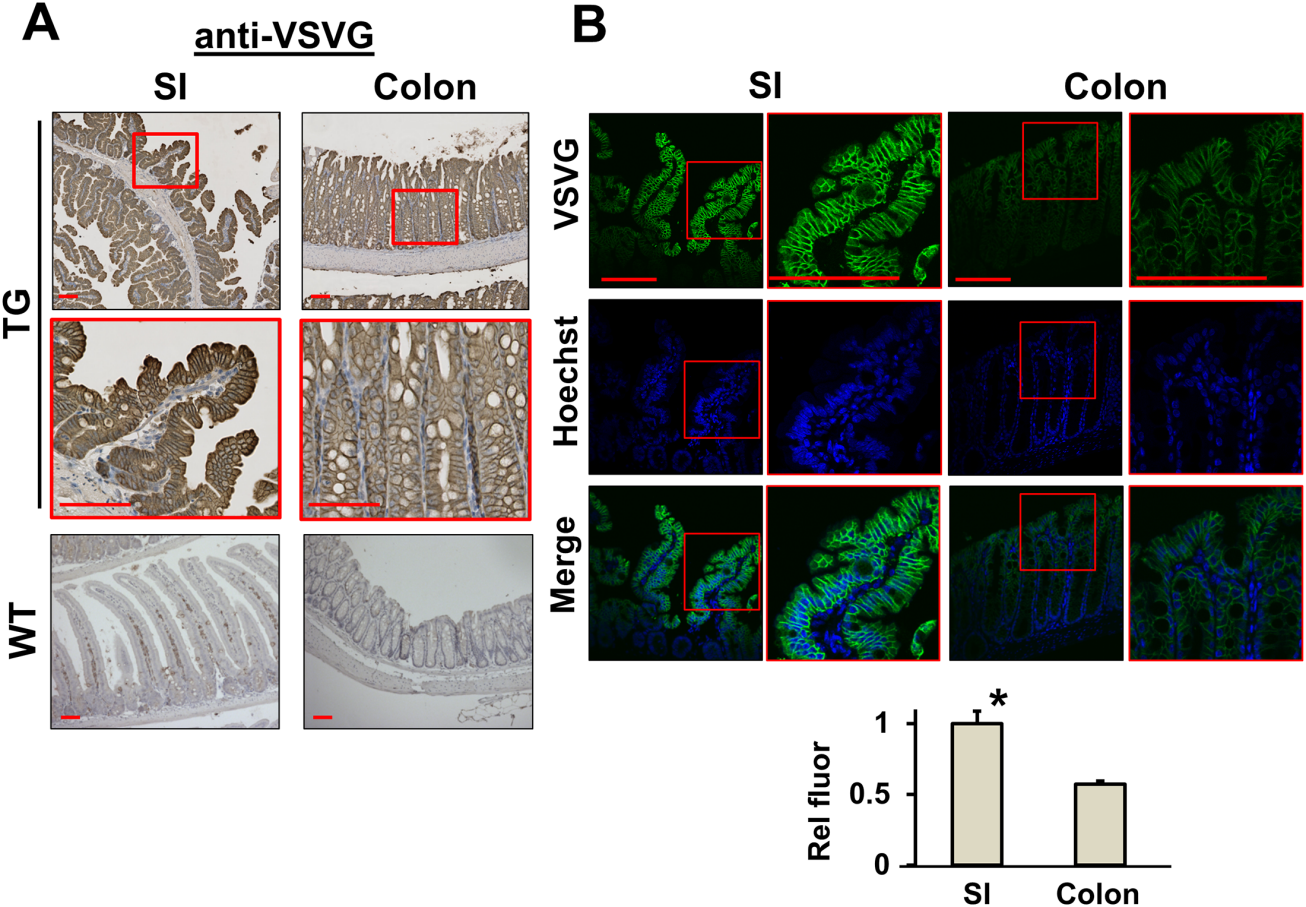
Expression of VSVG-LPA_2_ in mice. **(A)** TG VSVG-LPA_2_ expression in small intestine (SI, left) and colon (right) of LPA_2_ TG (upper two panels) and WT (lower panel) mice was determined. Anti-VSVG antibody was used for immunohistochemistry. Magnification: x100. Lower panels show magnified views (x400) of the boxed area. WT was used as a negative control. Bar: 100 μm. **(B)** VSVG-LPA_2_ expression in the small intestine and colon of TG mice was assessed by immunofluorescence microscopy. Bar: 100 μm. Images were captured under identical setting to compare the relative expression levels of VSVG-LPA_2_ in small intestine and colon. Integrated fluorescence signal intensity was averaged over the area of epithelial cell layer. Lower graph shows the relative fluorescence signal intensity. *, *p* < 0.01, small intestine versus colon. n = 3 per group. Statistical analysis was performed using 2-tailed Student’s t test.

The transgenic expression of VSVG-LPA_2_ was confirmed by immunohistochemistry analysis with anti-VSVG antibody. LPA_2_ expression was detected at the apical and basolateral membranes of the epithelium in both small intestine and colon (Fig 3A). Cytoplasmic staining of the anti-VSVG antibody was also observed, probably indicating internalized LPA_2_. Immunofluorescence analysis of small intestinal and colonic tissues using anti-VSVG antibody under the identical conditions showed that the fluorescence intensity of VSVG is about two-fold greater in the small intestine than in colon (Fig 3B).

### Altered IEC proliferation and differentiation in LPA_2_ transgenic mice

**Fig 4:**
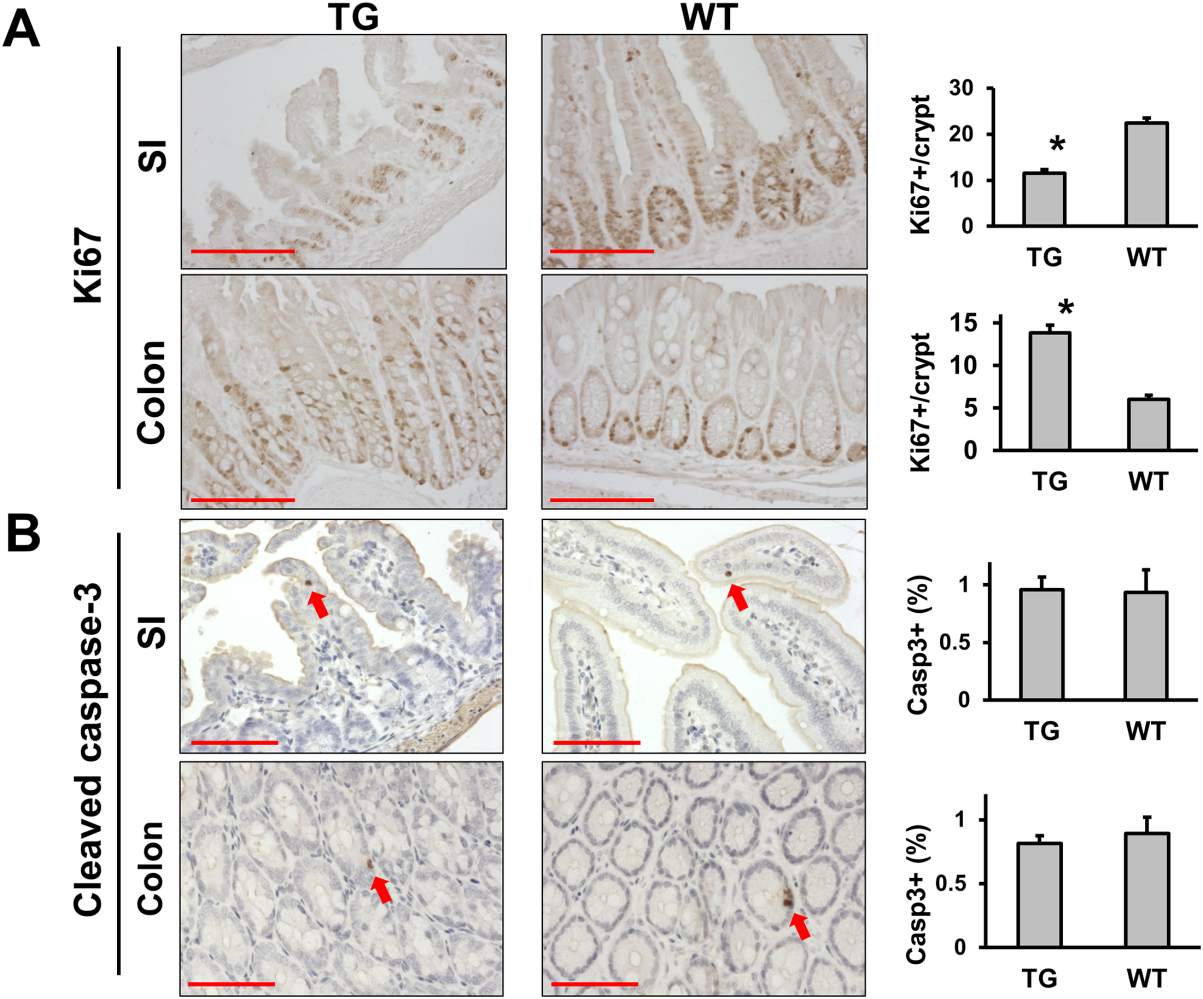
LPA_2_ overexpression results in opposite effects in the small intestine and colon. IEC proliferation **(A)** and apoptosis **(B)** were determined by immunohistochemical analysis for Ki67 and cleaved caspase-3, respectively. Representative images of TG and WT mice are shown Magnification: x200. Arrows point to apoptotic cells positive for cleaved caspase-3. Magnification: x200. Bar: 100 μm. *Right*, Ki67 proliferation and cleaved caspase-3 index are shown. The number of Ki67+ cells per crypt was quantified from thirty crypts per mouse. The level of apoptosis was expressed as percentage of apoptotic cells. Twenty fields of vison per mouse were counted. * *P* < 0.01. n = 3. Statistical analysis was performed using 2-tailed Student’s t test.

To get an insight into the cellular changes induced by the transgenic expression of LPA_2_, we initially determined cell proliferation and apoptosis by immunohistochemical staining of Ki67 and cleaved caspase-3, respectively. There was a significant decrease in Ki67-positive cells in the intestinal crypts of the transgenic mice compared with WT controls (11.5 ± 0.8 Ki67-positive cells/crypt in TG vs 22.4 ± 1.1 cells/crypt in WT, p < 0.01) (Fig 4A). Moreover, while Ki67-positive cells continuously labeled the crypts, Ki67-positive cells were spread unevenly with non-continuous presence of proliferating cells in transgenic crypts. This was a surprising outcome in light of previous studies that LPA_2_ induces cell proliferation [14]. In contrast to the small intestine, transgenic mice had increased numbers of proliferating cells in the colonic crypts than WT, and Ki67-positive cells extended to the middle of the crypts (13.8 ± 0.95 vs 6.0 ± 0.47, p<0.01), consistent with the hyperplasia in the colon. However, we did not observe a marked difference in the number of apoptotic cells between transgenic and WT mice (small intestine, 0.96 ± 0.11% of total cells for TG vs 0.93 ± 0.20% for WT; colon, 0.81 ± 0.06% for TG vs 0.89 ± 0.13% for WT) (Fig 4B).

**Fig 5:**
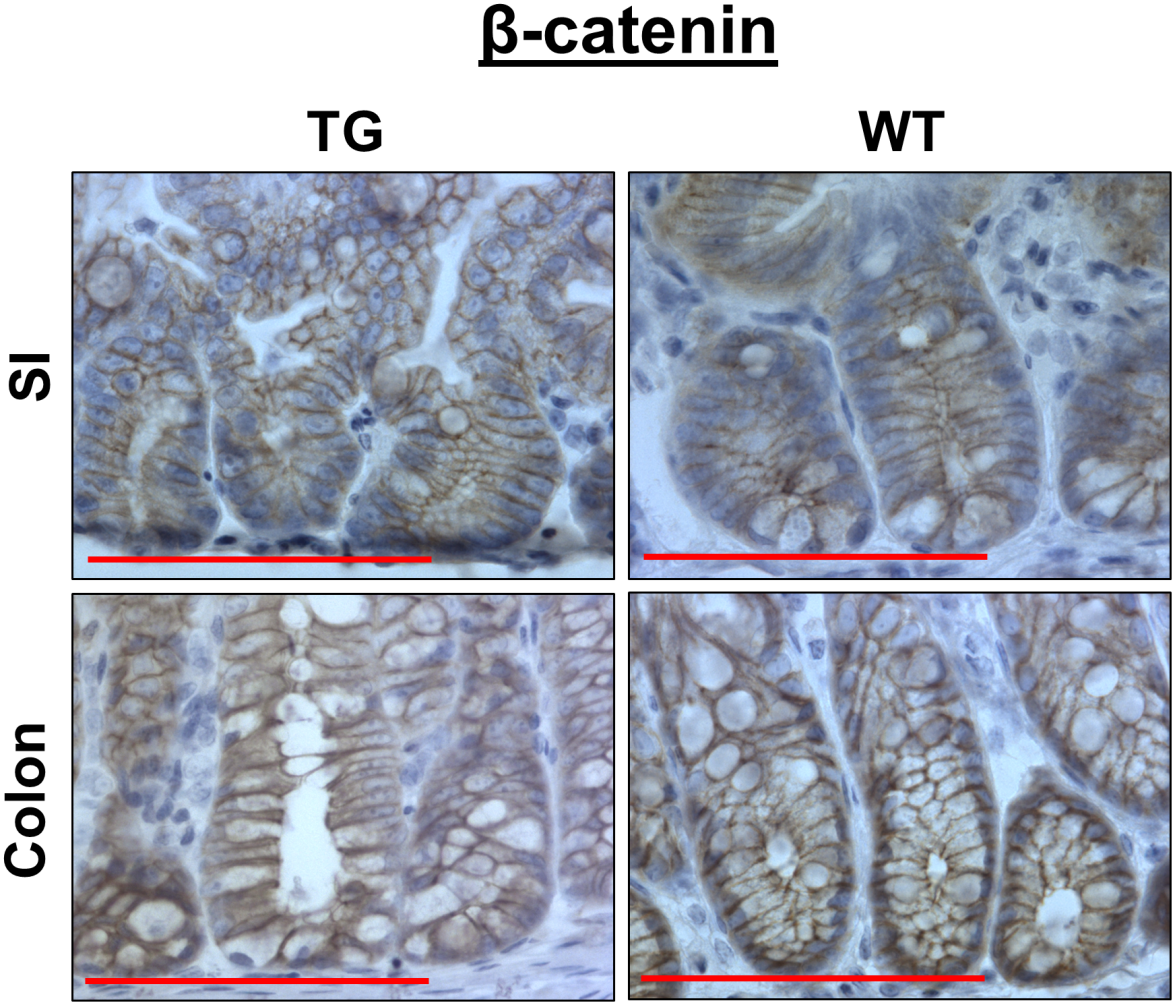
LPA_2_ overexpression does not affect β-catenin expression. β-catenin expression in the small intestine (SI) and colon was compared between TG and WT mice. Representative images of β-catenin staining are shown.Bar: 100 μm. n = 3.

Previous studies have shown that LPA_2_ activates β-catenin in colon cancer cells [13, 14, 17]. Hence, we examined whether β-catenin expression is altered by LPA_2_ transgenic expression. Unlike the observation in colon cancer cells, β-catenin expression in transgenic mouse was similar to that of WT controls (Fig 5). β-catenin was localized to the membrane and nuclear β-catenin was hardly evident. These results suggest that although LPA_2_ enhances β-catenin phosphorylation in colon cancer cells that harbor a mutation in the Wnt/β-catenin pathway, LPA_2_ alone is not sufficient to modulate β-catenin in the absence of a predisposing mutation in Wnt pathway.

**Fig 6:**
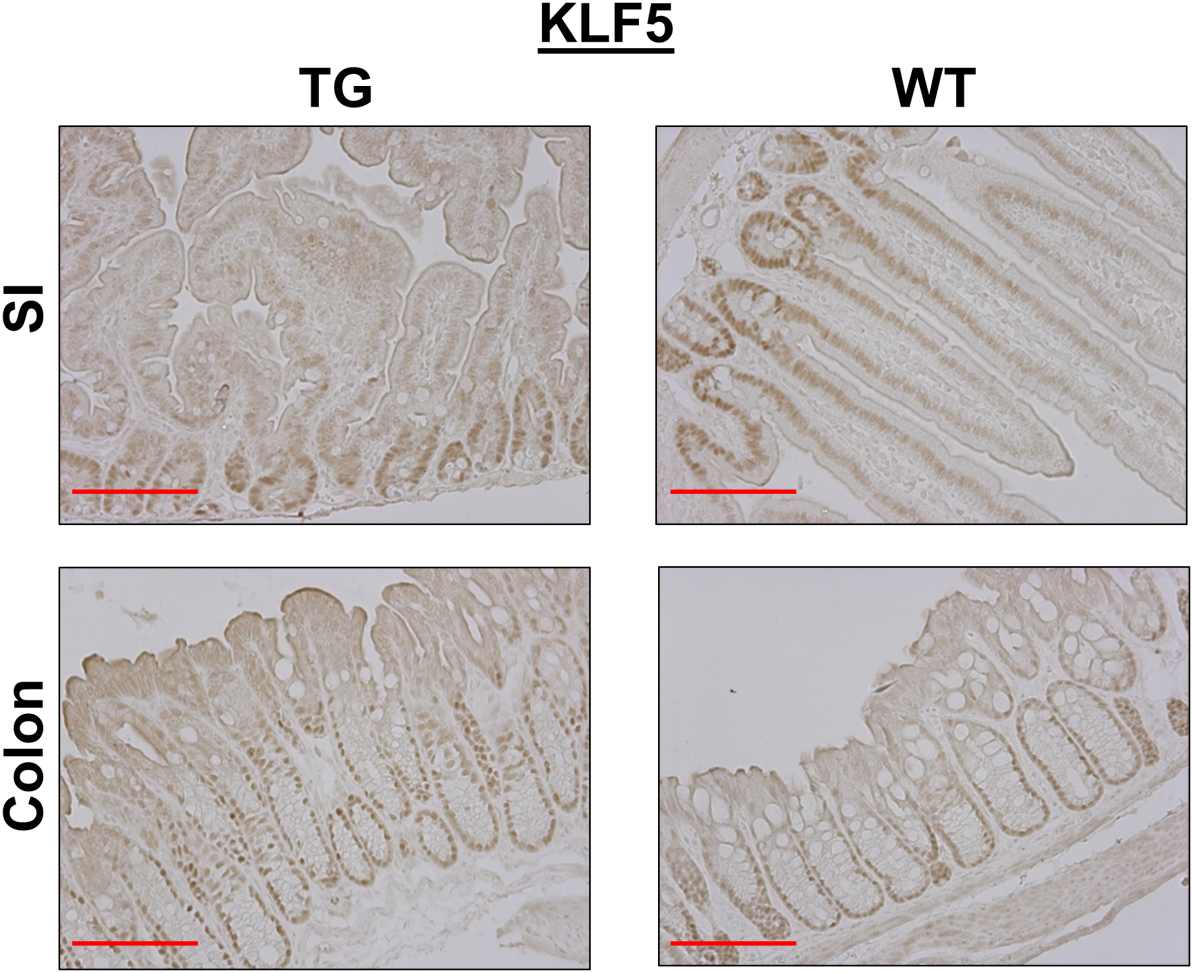
LPA_2_ overexpression results in opposite effects of KLF5 expression in the small intestine and colon. KLF5 expression in the small intestine (SI) and colon of TG and WT mice are shown. Magnification: x200. Bar: 100 μm. n = 3.

KLF5, a transcription factor enriched in proliferating cells in the crypt compartment of the intestine, is another target gene regulated by a LPA_2_-dependent mechanism [14, 24]. Immunohistochemical analysis showed the expression of KLF5 in the crypt compartments of WT small intestine with a gradual decrease toward the villus tip (Fig 6). In comparison, KLF5 expression was confined to the crypt regions of transgenic mice with infrequent presence above the crypts. In the colon, KLF5 expression extended past the mid-crypt region compared with WT colon. Together, these results suggested that overexpression of LPA_2_ is sufficient to promote IEC proliferation in the colon, but LPA_2_ overexpression results in dysplasia and inhibits proliferation of IECs in the small intestine.

**Fig 7:**
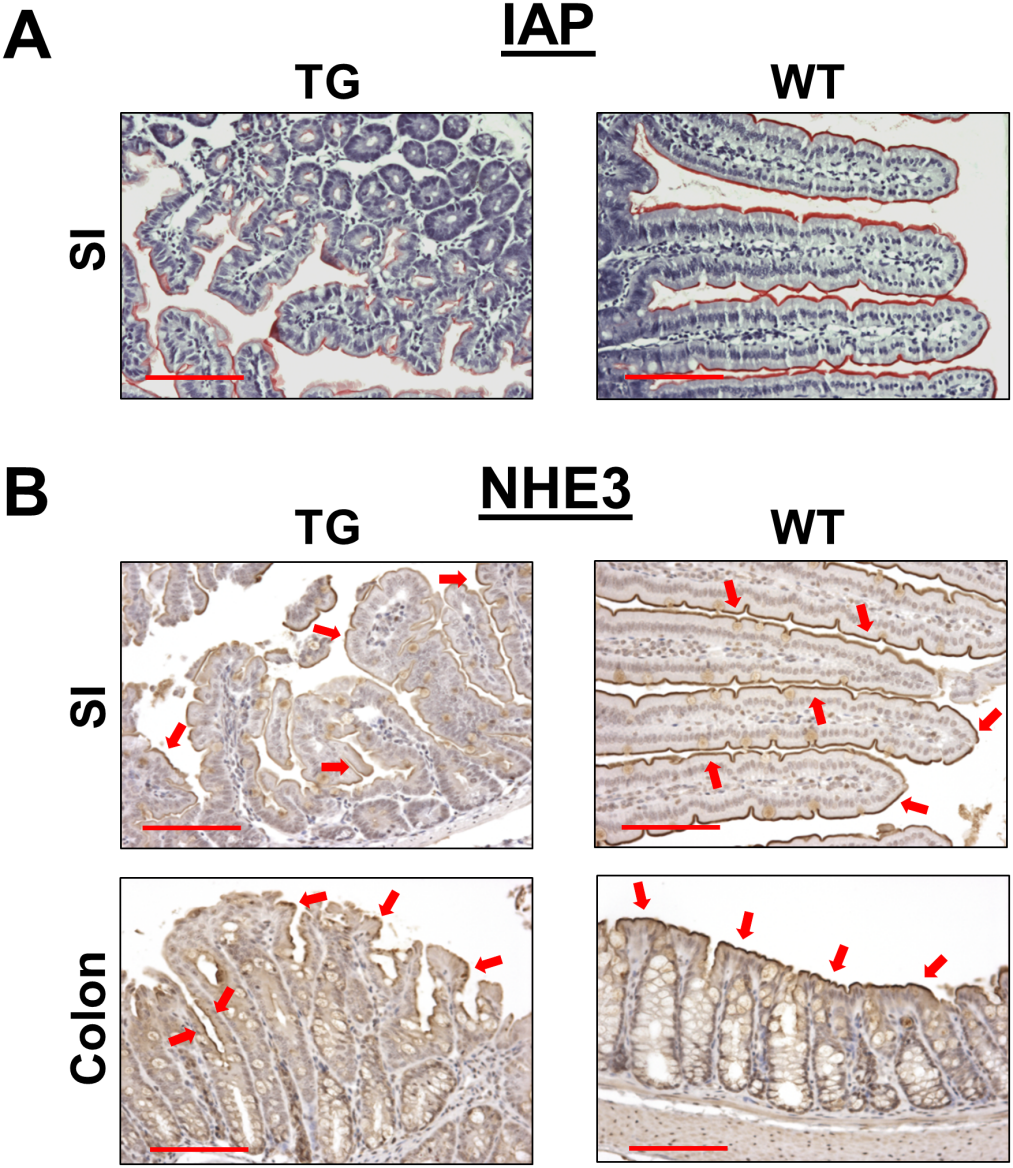
Absorptive epithelial cells in LPA_2_ TG intestinal tract are poorly differentiated. **(A)** IAP staining in the small intestine was determined by a chromogenic assay. Red color indicates IAP activity. Magnification: x200. Bar: 100 μm. **(B)** Immunohistochemically staining of NHE3 on the surface membrane of the small intestine and colon is shown. In comparison to WT, NHE3 staining in TG intestine was discontinuous and less uniform. Arrows mark NHE3 label on the membrane. Magnification: x200. Bar: 100 μm.

Dysplastic appearance of the intestinal epithelium suggests that the differentiation of IECs might be altered by overexpression of LPA_2_, which then contributes to intestinal dysfunction. To investigate the terminal differentiation of IECs in TG mice, we determined intestinal alkaline phosphatase (IAP) expression. Fig 7A shows reduced IAP activity in the small intestinal brush border membrane of TG mice compared with WT control. The IAP gene is only expressed in differentiated enterocytes of the small intestine, and IEC differentiation was further assessed by immunohistochemical analysis of Na^+^/H^+^ exchanger 3 (NHE3), which is the apical membrane transporter that mediates sodium absorption across the luminal surface of the small intestine and proximal colon [25]. Consistent with reduced IAP activity, NHE3 expression was markedly decreased in LPA_2_ TG intestine. Additionally, NHE3 staining was reduced and discontinuous in much of the luminal surface of colon (Fig 7B).

**Fig 8:**
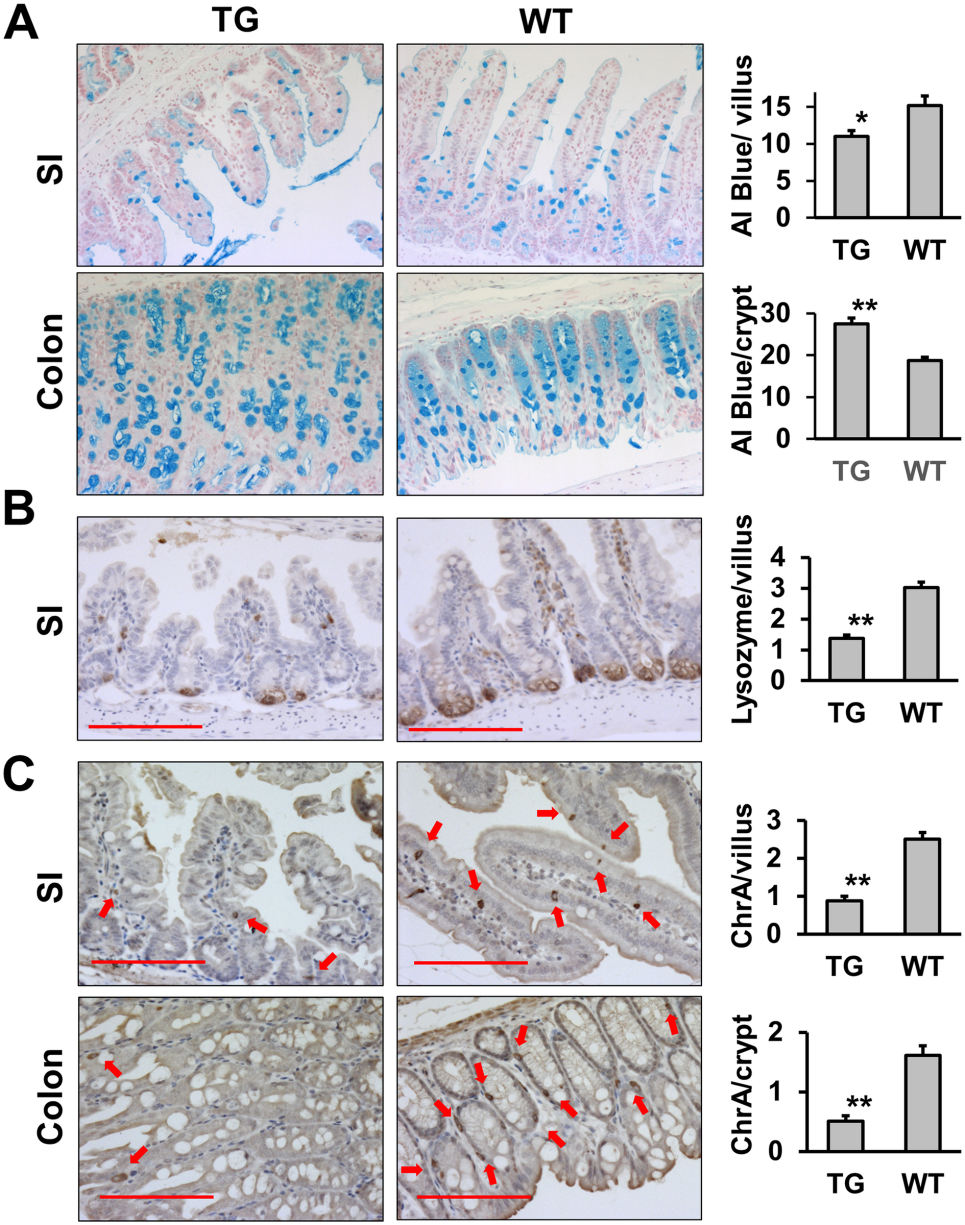
Decreased number of Paneth cells and enteroendocrine cells in LPA_2_ TG intestinal tract. Goblet **(A)**, Paneth **(B)**, and enteroendocrine **(C)** cells were identified by Alcian blue, lysozyme, and Chromogranin A expression, respectively. Magnification: x200. Bar: 100μm. Arrow, enteroendocrine cells. Goblet, Paneth, and enteroendocrine cells were quantified by counting alcian blue-positive, lysozyme-positive, and ChrA-positive cells per villus or crypt, respectively. Fifty villi or crypts were quantified for each mouse. The graphs show the number of each cell type (mean ± SEM). Statistical analysis was performed using 2-tailed Student’s t test. * *P* < 0.05, ** *P* < 0.01.

Since the terminal differentiation of absorptive cells is altered in the LPA_2_ TG intestine, we next determined whether secretory cell lineage is altered. Fig 8 depicts representative staining of alcian blue (goblet cells, Fig 8A), lysozyme (Paneth cells, Fig 8B), and CgA (enteroendocrine cells, Fig 8C). Goblet cell population in TG small intestine was significantly lower compared with WT (11.0 ± 0.83 cells vs 15.2 ± 1.31 cells per villus, p<0.05). On the other hand, there was increased number of goblet cells in the TG colon (27.5 ± 1.34 cells vs 18.7 ± 0.80 cells per crypt, p<0.01). The number of Paneth and enteroendocrine cells was reduced in TG intestinal tract.

### Correlation between LPA_2_ TG expression and dysplasia in the founder mice

**Fig 9:**
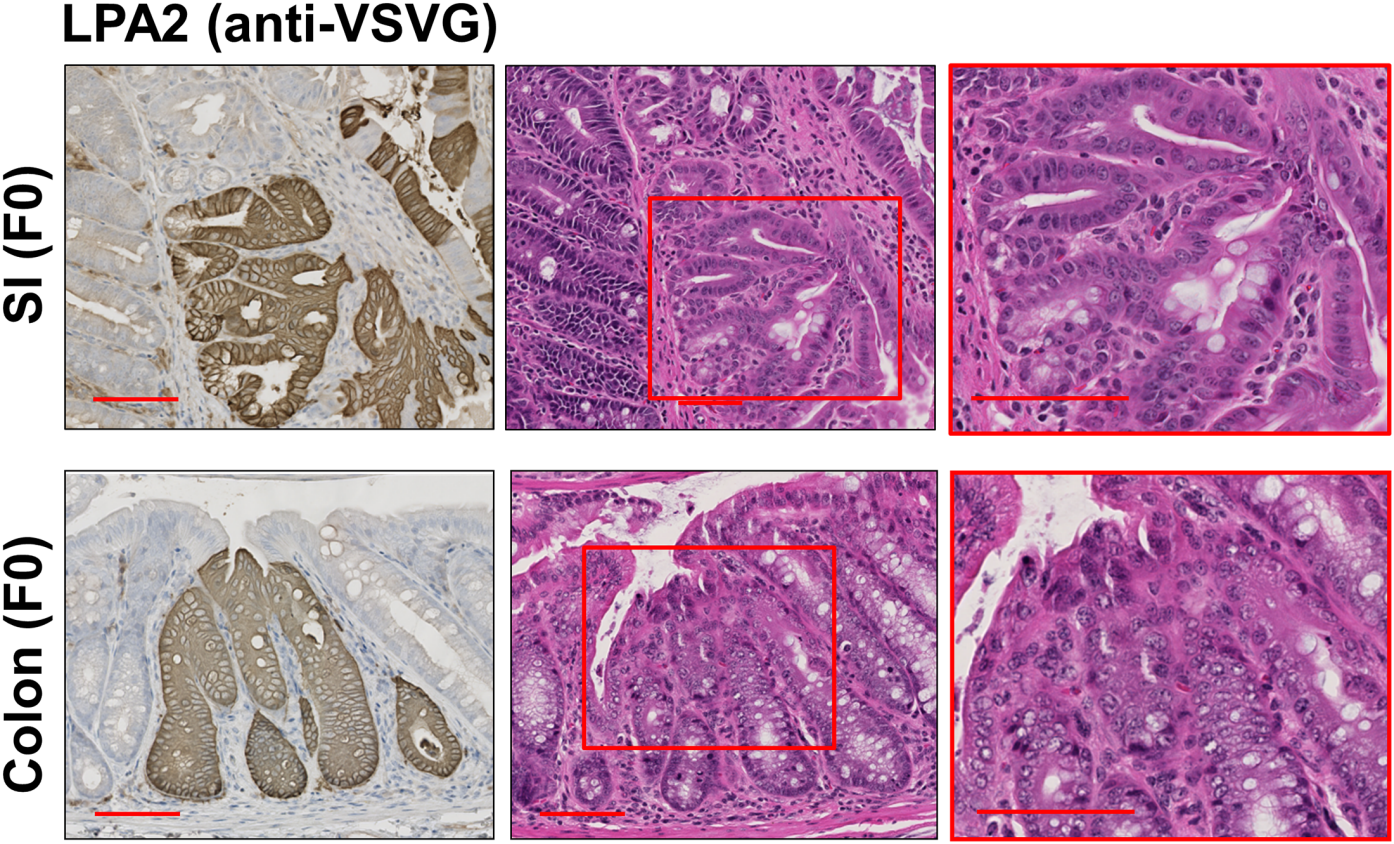
LPA_2_ overexpression correlates with the occurrence of dysplasia in founder mice. TG VSVG-LPA_2_ expression was determined in founder mouse (F0) small intestine and colon. Left panels show representative IHC staining using anti-VSVG antibody. Right panels show H&E of serial sections. Magnification: x200. Insets show a magnified view (x400) of the boxed area. Bar: 100 μm. n = 4.

The findings in transgenic mice suggest that the defective IECs are likely cause of early mortality of transgenic mice and may also explain the low birth rate of transgenic mice such that some fetus with germline transmission might have died in the womb. Although the founders appeared normal, the founders were not as fertile as WT mice with a longer duration between impregnation and infertility in males. Although the extent of LPA_2_ varied between individual founder mice, all the animals showed the mosaic pattern of transgenic LPA_2_ expression in the intestinal tract (Fig 9). Remarkably, some areas with transgenic LPA_2_ expression in both small intestine and colon showed epithelial dysplasia. These observations confirm that transgenic LPA_2_ expression is a direct cause of intestinal dysplasia.

## Discussion

We have reported the role of LPA_2_ on tumor progression such that the absence of LPA_2_ significantly decreases tumor burden in the *Apc*^*Min*^ and colitis-associated tumor models of colorectal cancer [10, 18]. Therefore, we hypothesized that TG expression of LPA_2_ in IEC promotes epithelial cell proliferation while decreasing apoptosis throughout the intestinal tract where the villin promoter drives human LPA_2_ expression. TG expression of human LPA_2_ in the colon resulted in hyper-proliferation and thickening of the crypt depth. Consistently, increased number of Ki67-positive cells was observed in the colon. Surprisingly, we found blunting and severe dysplasia of villi in the small intestine, where cell proliferation was reduced. The underpinning reason for the discrepancy is not known, but immunofluorescence analysis for VSVG-LPA_2_ expression showed that the expression levels of TG LPA_2_ is about two-fold greater in the small intestine. Villin promoter activity and its expression are greater in the small intestine than in colon [26, 27]. LPA is known to regulate actin cytoskeletal dynamics, which is closely associated with cell division [28]. Hence, one possibility is that increased LPA_2_ expression in the small intestine might alter the rate of cell division through dysregulation of actin cytoskeleton. Another possibility is that dysplasia in small intestinal epithelia negatively alters cell division. Future investigation is needed to resolve these possibilities.

LPA_2_ promotes proliferation of human colon cancer cell lines, such as HCT116 and LS174T, which harbor a mutation in β-catenin [13, 14, 17]. Consistently, the absence of LPA_2_ decreased the activation of β-catenin in the tumors of *Apc*^*Min/+*^ mice and azoxymethane/dextran sulfate sodium model of colorectal cancer [10, 18]. Hence, our current observation that TG LPA_2_ did not alter β-catenin in IECs suggests that overexpression of LPA_2_ alone is insufficient for activation of β-catenin. Current study is in an agreement with the earlier report by us that the activation of β-catenin in normal-looking IECs of *Apc*^*Min/+*^ mice was not affected by the loss of LPA_2_ [10]. Together, these studies imply that the LPA-LPA_2_ cascade does not invariably regulate β-catenin, but rather is dependent on a condition precedent of transformation that is present in human colon cancer cell lines and adenomatous lesions of Apc^Min/+^ mice.

Similarly to β-catenin, we observed no significant shift in IEC apoptosis by TG expression of LPA_2_. This may appear contradictory to the previous studies [11, 12], but the absence of LPA_2_ does not alter IEC apoptosis, indicating that LPA_2_ does not modulate the survival of IECs under basal conditions.

LPA_2_ TG mice were not born in a Mendelian proportion. Out of more than 70 F1 offsprings, only 3 had germline transmission of the transgene, indicating that either the villin promoter-driven transgene was not amenable to germline transmission or the LPA_2_ transgene caused embryonic morbidity. The former is unlikely based on previous IEC-specific gene expression using the same promoter [26, 27, 29]. The latter possibility, on the other hand, is in part supported by the presence of dysplasia in the founder mice. Although the expression of transgenic LPA_2_ did not always result in dysplasia, the overlap between transgenic LPA_2_ expression and the presence of dysplasia is clearly noteworthy. The low frequency of germline transmission of TG LPA_2_ indicates that overexpression of LPA_2_ in the intestinal tract obstructs normal embryonic development. Moreover, three transgenic mice as F1 were frail and had hematochezia, indicating intestinal dysfunction. The latter assumption was further supported by decreased IAP and NHE3 expression in the apical membrane of IECs. IAP is a brush border membrane protein, whose expression in in associated with intestinal epithelial cell differentiation [30]. Similarly, NHE3 is expressed on the apical surface of absorptive epithelial cells along the villus-crypt axis of the small intestine, and on the luminal surface of the colon [25]. In TG mice, expression of both IAP and NHE3 was discontinuous along the luminal surface, which implies that terminal differentiation of surface epithelial cells was disrupted, thereby compromising absorptive functions. It has been reported that LPA modulates cortical neuroblast morphology and myeloid differentiation [31, 32], but LPA_2_-deficiency does not show a significant effect on mouse intestinal morphology [10, 18]. Interestingly, TG LPA_2_ decreased the number of secretory cells with an exception of goblet cell numbers in the TG colon. The reason for the opposite effect on goblet cells in the small intestine and colon is not clear. Although the increase in goblet cells in the TG colon correlated with hyperplasia, a parallel increase in enteroendocrine cell population was not observed, suggesting that hyperplasia alone cannot account for the increased goblet cell number in the TG colon. We suspect that the dysplastic transformation by LPA_2_ overexpression, rather than a direct effect on secretory lineage, is the cause of the changes in TG mice. The specific effect of LPA on cell lineages within the intestinal tract requires further investigations using perhaps a more malleable model such as an inducible TG expression of LPA_2_.

In summary, we demonstrate that overexpression of LPA_2_ induces dysplasia in mouse intestine that alter IEC proliferation and differentiation. Although LPA_2_ overexpression did result in intestinal malignant transformation, our results reinforce the importance of the LPA-LPA_2_ axis in homeostatic regulation of IECs and its potential contribution to carcinogenesis in the intestinal tract.
